# Medications and Supplements Associated With Drug-Induced Glaucoma

**DOI:** 10.7759/cureus.110497

**Published:** 2026-06-08

**Authors:** Tarek Hammadieh, Ali Istanaksai, Khaled Jama, Abdalla Ali, Eesa Siddiqui, Haidar Bilal

**Affiliations:** 1 Medicine, Epsom Hospital, Epsom, GBR; 2 Medicine, North Middlesex University Hospital, London, GBR; 3 Psychiatry, St George's Hospital, London, GBR; 4 General Surgery, Whittington Hospital, London, GBR; 5 Medicine, Royal Free Hospital, London, GBR; 6 Elderly Medicine, Epsom Hospital, Epsom, GBR

**Keywords:** acute angle-closure glaucoma, drug-induced glaucoma, intraocular pressure (iop), open-angle glaucoma, systemic medication

## Abstract

Glaucoma is a common condition that damages the optic nerve, usually as a result of an increase in intraocular pressure (IOP). It can be divided into two types: open-angle glaucoma (OAG) and acute angle-closure glaucoma (AACG). OAG is believed to occur due to dysfunction in aqueous outflow through the trabecular meshwork (TM) of the eye as it flows from the posterior to the anterior chamber. AACG manifests when a bulging iris blocks the iridocorneal angle, commonly caused by pupillary block due to pupil dilation, leading to fluid buildup and increased IOP, potentially resulting in complete vision loss. As a result of an aging population, chronic conditions are increasing in number, necessitating the use of systemic medications to treat them. However, many of these medications may affect the eye, predisposing it to different ocular morbidities. This narrative review will examine the effects of these medications on glaucoma development, specifically focusing on the effects of corticosteroids on OAG through increased aqueous humor resistance in the TM and the effects of sulfonamides, anticholinergics, antidepressants, cholinergics, anticoagulants, and certain supplements on AACG as a result of their sympathomimetic properties and the disruption of the lens-iris structure.

## Introduction and background

Glaucoma is the most common cause of irreversible blindness worldwide. It can be classified into primary and secondary based on etiology and into open-angle glaucoma (OAG) and acute angle-closure glaucoma (AACG) based on the iridocorneal angle [1]. The estimated prevalence of glaucoma globally was 76 million in 2020 and is expected to reach 111.8 million in 2040 [2]. Therefore, greater emphasis must be placed on understanding the causes of this condition to prevent its incidence from rising further.

OAG is believed to occur as a result of intraocular fluid buildup secondary to increased drainage resistance from a faulty or inefficient drainage system of the trabecular meshwork (TM). This could be due to partial obstruction from foreign material, a reduction in the number of trabecular endothelial cells, a decreased density of trabecular pores, the number of vacuoles, or the size of the inner wall endothelium of the canal of Schlemm, loss of phagocytic activity, or dysfunction in the neurological feedback loop involved in the drainage of aqueous humor. This fluid accumulation increases intraocular pressure (IOP), which can damage the optic nerve over time via vascular dysfunction, leading to ischemia and mechanical compression of axons. This results in progressive peripheral visual field loss, followed by central vision impairment [3].

The main risk factors for OAG are high IOP, age, family history, and ethnicity, with the incidence being three to four times higher in African American individuals [4]. AACG is more serious and can result in blindness if not treated quickly. There are many causes of this type of glaucoma, but the most common cause is pupillary block due to pupil dilation. This happens when the pupil merges with the lens, preventing aqueous humor from flowing into the anterior chamber, leading to a rise in pressure in the posterior chamber [5]. Other causes of AACG include plateau iris, choroidal effusion, aqueous misdirection, and posterior segment tumors. Plateau iris consists of a normal central anterior chamber depth, a flat iris profile, and crowding of the angle by the iris base. Angle closure occurs due to forward displacement of the iris base by the ciliary process [6]. Suprachoroidal effusion narrows the iridocorneal angle by forward displacement of the lens-iris diaphragm. Risk factors for AACG are advanced age, female sex, family history, hypermetropia, and Asian ethnicity. Symptoms to look out for include elevated IOP, blurry vision, nausea, headache, and halos around light.

Although certain risk factors may naturally increase the chance of glaucoma development, specific medications are known to accelerate the risk through many different mechanisms. Corticosteroids commonly give rise to OAG by increasing aqueous flow resistance in the TM due to increased deposition and decreased clearance of the extracellular matrix when the glucocorticoid receptor (GR) is stimulated [1].

However, most medications known to influence the development of glaucoma, through different mechanisms, are linked specifically with AACG. These medications may precipitate an attack in populations with a narrow iridocorneal angle [7]. Sulfonamides, cholinergic agents, and anticoagulants induce angle closure by non-pupillary block mechanisms through choroidal effusion, miosis, and suprachoroidal hemorrhage, respectively (Table 1). Conversely, anticholinergics, serotonergics, and anticoagulants trigger angle closure by pupillary block through their sympathomimetic/parasympatholytic properties in individuals with a narrow iridocorneal angle [1].

**Table 1:** Summary of medications associated with AACG AACG, acute angle-closure glaucoma

Medication class	Examples	Mechanism of angle closure	Ocular effects
Sulfonamides	Topiramate, acetazolamide, and methylsulfonylmethane supplement	Anterior displacement of the lens-iris diaphragm	Ciliochoroidal effusion
Anticholinergics	Atropine, glycopyrrolate, oxybutynin, scopolamine, and botulinum toxin	Pupillary block	Mydriasis and cycloplegia
Serotonergics	Venlafaxine, citalopram, triptans, and imipramine	Pupillary block	Mydriasis, increased aqueous humor production, and uveal effusion
Cholinergics	Pilocarpine	Anterior displacement of the lens-iris diaphragm	Miosis and anterior rotation of the lens-iris diaphragm
Anticoagulants	Warfarin, apixaban, and heparin	Anterior displacement of the lens-iris diaphragm	Suprachoroidal hemorrhage

Despite being fairly uncommon overall, the adverse reactions caused by drug-induced glaucoma can be sight-threatening and can result in severe ocular morbidity if not recognized quickly. Therefore, an improved understanding of these medications, along with their clinical implications, may help reduce the increasing incidence of drug-induced glaucoma. This narrative review aims to summarize the current evidence regarding drug-induced glaucoma. Owing to the heterogeneity of the available literature, findings are presented descriptively rather than through quantitative meta-analysis.

## Review

Corticosteroids

Steroids are popular medications that are prescribed to treat a range of autoimmune and inflammatory conditions, including rheumatoid arthritis, systemic lupus erythematosus, inflammatory bowel disease, and congenital adrenal hyperplasia [8]. Despite offering various benefits, steroids can induce adverse effects throughout the body, including the eye, where they have been known to play a role in the development of cataracts and glaucoma [9]. They function by binding to GRs, ligand-activated transcription factors, in the cytoplasm of cells, inhibiting the genes encoding inflammatory leukocytes and structural cells and therefore exerting anti-inflammatory and immunosuppressive effects [10]. The route of corticosteroid administration depends on the disorder being treated. They can be parenteral, oral, inhaled, or topical.

Steroid-induced glaucoma (SIG) is classified as secondary OAG, where it is thought to occur as a result of an increase in resistance to aqueous flow in the TM. The increased production and decreased clearance of the extracellular matrix of the TM are thought to cause this, whereby there is an abundance of glycosaminoglycans, fibronectin, elastin, and type IV collagen and a reduced activity of matrix metalloproteinases [11]. The GRs in TM cells are influenced by steroids, affecting cell migration and phagocytosis. Specifically, GR beta is known to block glucocorticoid responsiveness in TM cells and inhibit phagocytic activity [12]. This interaction causes an increase in extracellular matrix deposition and decreases the cellularity of the TM, further increasing aqueous outflow resistance and raising IOP. Over time, the raised IOP may accelerate glaucoma development when the value is high enough, facilitating optic nerve damage and visual field defects. A study in which dexamethasone mini-pumps were implanted in mice and monitored weekly showed that IOP increased by 2.6 ± 1.6 mmHg (mean ± SD) after three to four weeks, with reduced conventional outflow facility by 52% compared with the control [13]. This study demonstrated that IOP is also increased in mice after steroid administration, mimicking the effect in humans and providing further evidence of the correlation between ocular hypertension and steroids. In another study, Becker and Mills noticed that patients with glaucoma and patients suspected of having glaucoma showed a substantial increase in IOP following treatment with topical corticosteroids for a few weeks [14].

Patients with a previous history of glaucoma have a higher chance of developing SIG after corticosteroid use. Acute and chronic increases in IOP can occur after steroid use, where the degree of severity depends on the dosage used. Studies have recorded increases in IOP within three to six weeks after the commencement of topical eye drop therapy, suggesting that steroids can play a role in raising IOP [15].

Sulfonamides

Sulfonamides are types of medications containing a sulfur functional group in their chemical structure. Sulfa-based medications are used to combat bacterial agents due to their antifolic properties but are also used as antidiuretics, antidiabetics, anticonvulsants, and more [16]. Sulfonamides work as antagonists of p-aminobenzoic acid in the enzyme dihydropteroate synthetase, which is essential for the production of folic acid needed for DNA replication. This therefore inhibits the production of dihydrofolate and tetrahydrofolate, bringing cell division and DNA growth to a halt [17]. Sulfa drugs tend to give rise to AACG, in particular topiramate and, to a lesser extent, acetazolamide, trimethoprim-sulfamethoxazole, zonisamide, chlorthalidone, and hydrochlorothiazide [18].

The exact mechanism by which sulfonamides induce AACG remains unclear; however, in contrast to corticosteroids, they exert their effects through non-pupillary block mechanisms. One theory suggests that osmotic imbalances within the crystalline lens cause hydration of the lens. As the lens enlarges, it displaces the lens-iris diaphragm anteriorly, and the anterior chamber becomes more shallow and increasingly susceptible to angle closure [19]. Another theory implies that sulfa-based medications give rise to ciliary body edema followed by supraciliary effusion, which causes the ciliary body to rotate forward, forming angle closure [20]. Other mechanisms include retinal edema and secondary shallowing of the anterior chamber linked with increased IOP [1]. AACG has been shown to resolve once topiramate was discontinued in two separate cases [21,22]. In both cases, the patients recovered from AACG symptoms a short while after the medication was discontinued, implying that topiramate contributed to the rise in IOP in those patients prior to discontinuation of the medication. Despite being used to lower IOP in acute cases of glaucoma, acetazolamide is another sulfonamide known to induce AACG. This has been demonstrated in a case in which a patient developed bilateral AACG with ciliary body edema as well as massive choroidal effusion with posterior retinal folds and papillary edema after administering acetazolamide following cataract surgery [23]. In another similar study, methazolamide induced acute myopia and AACG with bilateral anterior chamber shallowing. This was associated with ciliary body edema and supraciliary effusions [24]. In both cases, the patients’ vision improved significantly a few days later, once the medication was discontinued, highlighting the impact that those drugs had on IOP and AACG development.

As mentioned in the previous studies, AACG precipitated by sulfa-based agents can be treated quickly by discontinuing the causative agent. The IOP should fall to normal levels once the offending medication is removed. It is believed that uveal effusions induced by sulfa-based drugs have an underlying inflammatory process that gives rise to AACG. Therefore, oral or intravenous corticosteroids can be beneficial given their anti-inflammatory properties [25]. Laser iridotomy has been shown to have no effect, as sulfonamide-induced AACG is not caused by pupillary block; however, ciliochoroidal effusion drainage has successfully been shown to restore anterior chamber depth and reduce IOP to baseline levels [26].

Anticholinergics

Anticholinergics have a diverse range of applications in the medical field. This drug group can be used to alleviate Parkinson’s symptoms, motion sickness/nausea, bronchoconstriction, and many other conditions. They exert their effect by antagonizing parasympathetic muscarinic acetylcholine receptors and therefore inhibiting the parasympathetic nervous system (PNS). Functions influenced by the PNS include involuntary actions of the smooth muscles in the lungs, urinary system, and GI tract [27]. In the eyes, they are responsible for mydriasis by binding to acetylcholine receptors on the iris sphincter muscle and subsequently leading to iridocorneal angle closure. Common anticholinergics include atropine, benztropine, glycopyrrolate, ipratropium, oxybutynin, and scopolamine [18].

Anticholinergics used for different purposes seem to have a noteworthy effect on the development of closed-angle glaucoma. Atropine, tropicamide, and cyclopentolate are common medications used before a fundoscopic examination to induce mydriasis. The risk of developing AACG after diagnostic mydriasis is reported to be three in 10,000 patients [28]. Nebulized ipratropium bromide is a common anticholinergic used alongside salbutamol to alleviate symptoms of acute asthma and exacerbation of chronic obstructive pulmonary disease. Angle closure as a result of ipratropium occurs when the agent escapes from the oxygen mask and diffuses into the cornea, causing pupillary dilation followed by pupillary block [5]. Unlike sulfonamides, which precipitate AACG through non-pupillary mechanisms, ipratropium induces AACG by causing semidilation of the pupil. This partially obstructs the flow of aqueous humor from the posterior to the anterior chamber, resulting in anterior displacement of the iris and subsequent occlusion of the drainage angle. Salbutamol aggravates the condition further through its sympathomimetic properties by increasing the production of aqueous humor and consequently raising the IOP. Case reports conducted by Shah et al., consisting of five patients who were all given ipratropium following acute exacerbation of chronic obstructive airway disease, described the development of AACG, highlighting the impact that ipratropium has on AACG formation [29].

Botulinum toxin is another anticholinergic that can elicit the development of angle closure. It works by suppressing the release of acetylcholine at the skeletal muscle neuromuscular junction, leading to temporary muscle paresis [25]. The toxin also induces ptosis and pupillary dilation when administered periorbitally. It induces these changes by traveling to the ciliary ganglion and inhibiting its activity or by exerting its effect on the pupillary sphincter muscle of the iris. This was evident in a case in which botulinum toxin was given for blepharospasm to a patient with a history of retinitis pigmentosa. A few weeks later, the patient was diagnosed with AACG with an IOP of 52 mmHg [30].

Glycopyrrolate is an antimuscarinic agent that reduces airway secretions as well as inhibits the muscarinic effects of neostigmine during neuromuscular blockade (NMB) reversal. It has been reported in a study that glycopyrrolate precipitated the development of AACG 12 hours after use to counteract NMB after surgery. In this case, the patient was placed in the prone position for 5.5 hours for a cervical spine procedure [31]. Although the patient was mydriatic and hyperopic, one may speculate that the increase in IOP may be due to the gravitational effect of the prone position.

Serotonergics

Serotonin (5-hydroxytryptamine (5-HT)) receptors are located throughout the central nervous system, GI tract, blood vessels, and the eye. Serotonergic drugs can be used to treat obsessive-compulsive disorder, depression, and other psychiatric conditions. They can be classified into selective serotonin reuptake inhibitors (SSRIs), serotonin and norepinephrine reuptake inhibitors (SNRIs), monoamine oxidase inhibitors, triptans, and tricyclic antidepressants. In the eye, 5-HT1A and 5-HT7 receptors are located on the choroid, ciliary body, iris sphincter muscle, and TM. Stimulating the 5-HT7 receptors potentiates mydriasis, relaxation of the iris sphincter, and aqueous humor production. SNRIs not only engage with 5-HT receptors but also stimulate adrenergic and dopaminergic receptors, resulting in mydriasis and increased aqueous humor production, respectively [32].

Despite being fairly uncommon, both short-term and long-term exposure to serotonergic medications have been associated with AACG. This was demonstrated in a study from Taiwan that found a 5.80-fold increased risk of developing AACG following daily SSRI use [33]. Older age and female sex also increase the risk of AACG. Although the specific mechanism by which these drugs influence AACG is not entirely clear, one suggested mechanism is that antidepressants exert a serotonergic effect by inducing angle closure through mydriasis and pupillary block. In addition, an increase in norepinephrine and serotonin levels elicited by SSRI and SNRI use can trigger pupil dilation by activating norepinephrine and 5-HT receptors [34]. Moreover, uveal effusion has been reported to be associated with AACG development after SSRI and SNRI use. The serotonin receptors present in the ciliary body and choroid are believed to be affected by serotonergic drugs, altering their metabolism and consequently giving rise to uveal effusion [35].

Interestingly, a study showed that AACG appeared to be triggered by two different classes of antidepressants in the same patient at different time points. Bilateral AACG with an IOP of 56 mmHg in the right eye and 34 mmHg in the left eye was triggered five months after citalopram was prescribed for depression. A few months later, the patient relapsed into angle closure two days after imipramine was administered, despite receiving iridotomies [36]. Another case study demonstrated the role of citalopram in the development of AACG, where a patient developed left angle-closure glaucoma following an overdose of 20 mg citalopram tablets [37]. Shortly after the overdose, the IOP in the left eye was reported to be 60 mmHg, in addition to left corneal edema and fixed dilated pupils. Although the mechanism of AACG occurrence appears to be unclear, there seems to be a direct association between SSRI use and angle-closure glaucoma in many of the studies discussed. It is also not certain that SSRIs were the cause of AACG development, and it could have been due to other factors that overlapped with SSRI use. Therefore, further research may be warranted to investigate the risk of development or progression of glaucoma in individuals with sustained serotonergic medication use.

Cholinergics

Cholinergic agents are a group of drugs that interact with acetylcholine, an important neurotransmitter within the PNS. They can be classified into two types based on how they work: direct-acting and indirect-acting. Direct-acting agents are agonists that directly bind to and stimulate muscarinic receptors and include acetylcholine, methacholine, muscarine, pilocarpine, and many more. Indirect agents work by causing a surge in acetylcholine at cholinergic receptors. Examples are physostigmine, neostigmine, tabun, and sarin. Cholinergics can be used in a wide range of medical conditions and are most notably used in patients with myasthenia gravis, dementia, and post-operative urinary retention [38].

In ophthalmology, cholinergics such as pilocarpine, acetylcholine, and carbachol can be used for pupil constriction in intraocular surgery and glaucoma treatment due to their miotic effect. They induce miosis by activating the iris sphincter muscle, allowing the iris to pull away from the TM. This can be used in the treatment of AACG caused by mydriasis, pupillary block, and closure of the iridocorneal angle [39]. However, despite this, cholinergics can also oppose this effect and cause AACG. Agents such as pilocarpine can exacerbate pupillary block by increasing contact between the iris and lens. In addition, cholinergic agents stimulate ciliary muscle contraction, leading to looser zonules, lens rounding, and lens displacement into the anterior chamber, similar to the AACG mechanism induced by sulfonamides [40]. Pilocarpine has been shown to induce paradoxical AACG in patients with spherophakia, phacomorphic glaucoma, exfoliation syndrome, and in patients without ocular morbidity. A specific study demonstrates this, whereby a patient with existing spherophakia developed acute angle closure with an IOP of 68 mmHg in her left eye 45 minutes after 2% pilocarpine was administered [41]. AACG has been observed in patients with specific risk factors such as spherophakia; however, there appears to be less emphasis placed on cholinergic-induced AACG in healthier patients with minimal risk factors. This could be an area for future exploration.

Anticoagulants

Anticoagulants are medications that inhibit blood clot formation and are prescribed to reduce the risk of conditions such as stroke, myocardial infarction, thrombosis, and embolism. They are classified according to their mechanism of action. Antiplatelets inhibit platelet function and platelet aggregation and include acetylsalicylic acid (aspirin), clopidogrel, dipyridamole, prasugrel, and ticagrelor. Oral anticoagulants act against clotting factors and include vitamin K antagonists, warfarin, and direct oral anticoagulants, such as apixaban (factor Xa inhibitor) and dabigatran (direct thrombin inhibitor). Heparin and fondaparinux are indirect factor Xa inhibitors that are administered subcutaneously or intravenously [42].

Anticoagulants are believed to cause AACG in elderly patients as a result of spontaneous suprachoroidal hemorrhage. Similar to cholinergics and sulfonamides, AACG is believed to be initiated by anterior mispositioning of the lens-iris diaphragm as a result of a large retinal and choroidal detachment [25]. In addition to older age and use of anticoagulants and thrombolytics, atherosclerosis and systemic hypertension are risk factors for this complication. Ocular morbidities, such as age-related macular degeneration with neovascularization and nanophthalmos, are also risk factors thought to play a role in suprachoroidal hemorrhages [1]. There are a handful of case reports that have demonstrated the association between anticoagulants and AACG [43-45]. In these studies, AACG was discovered in patients who take warfarin regularly despite having an INR within the normal therapeutic range. These case reports, therefore, convey that having an INR within the therapeutic range may not necessarily mean that the patient is safe from spontaneous hemorrhages and that physicians should look out for early signs to rule out this phenomenon. The patient in the last study also had a history of atrophic age-related macular degeneration, a known risk factor discussed earlier that could have precipitated the suprachoroidal hemorrhage [45].

Supplements

Supplements, such as vitamins and minerals, have been used alongside medical treatments and, in some cases, have even replaced them in many different illnesses. Some supplements are known to exert a beneficial effect intraocularly by reducing IOP and improving ocular blood flow. This is highlighted in a study that demonstrated that flavonoids improve ocular blood flow and forskolin lowers IOP significantly [46]. On the other hand, some supplements seem to have a negative effect on the eyes. Consuming large amounts of certain types of supplements has been associated with the development of glaucoma.

Calcium and iron can pose a slight threat to the eye by specifically playing a role in the development of glaucoma, most likely primary open-angle glaucoma (POAG). A sample consisting of a US-based population of adults over 40 years old demonstrated that consuming over 800 mg/day of calcium or over 18 mg/day of supplemental iron increased the likelihood of self-reported glaucoma compared with those who do not take them [47]. Calcium and iron are believed to play a role in the pathogenesis of neuronal cell death as well as aqueous humor outflow dysfunction due to their oxidant properties, enabling them to accelerate glaucoma occurrence. Caffeine is another factor associated with the development of glaucoma, as it is linked with a transient increase in IOP and therefore POAG. Although the specific mechanism is not yet known, it may facilitate this by increasing aqueous humor production and reducing outflow activity [48]. In patients with susceptible eyes, the rise in IOP may induce glaucoma formation. Prospective study data utilizing 79,120 women from the Nurses’ Health Study and 42,052 men from the Health Professionals Follow-up Study reported that caffeine consumption was not linked with the risk of developing POAG. However, the study reported an increased probability of developing POAG in individuals who consume caffeine and have a family history of POAG associated with an IOP increase [18].

Methylsulfonylmethane (MSM) is an antioxidative, anti-inflammatory, and antihistamine agent known to be used in detox products [49]. It has a sulfonyl group that is identical to the one found in sulfa-based agents. Similar to how topiramate induces AACG, MSM also leads to AACG by giving rise to uveal effusions and anterior rotation of the lens-iris diaphragm [50].

## Conclusions

Several medications can influence IOP and contribute to the development of both open-angle and angle-closure glaucoma through a variety of complex mechanisms. These mechanisms have enabled us to understand the interaction between these drugs and specific components of the eye and subsequently transition into a pathological state. Despite limited evidence of a direct association between these agents and glaucoma development, their effects on the eye are evident. Further research is required to strengthen the current evidence regarding the role of these medications in glaucoma development. A clearer understanding of their effects will enable clinicians to make more informed clinical decisions and better assess the associated risks.
